# Precise control of liposome size using characteristic time depends on solvent type and membrane properties

**DOI:** 10.1038/s41598-023-31895-z

**Published:** 2023-03-23

**Authors:** Sunghak Choi, Bongsu Kang, Eunhye Yang, Keesung Kim, Moon Kyu Kwak, Pahn-Shick Chang, Ho-Sup Jung

**Affiliations:** 1 Present Address: Center for Food and Bioconvergence, Department of Food Science and Biotechnology, Seoul National University, Seoul, 08826 South Korea; 2 School of Mechanical Engineering, Kyungpook National University, Daegu, 41566 South Korea; 3 Department of Agricultural Biotechnology, Seoul National University, Seoul, 08826 Republic of Korea; 4 Research Inst. of Advanced. Materials, Collage of Engineering, Seoul National University, Seoul, 08826 South Korea; 5 Center for Agricultural Microorganism and Enzyme, Seoul National University, Seoul, 08826 Republic of Korea; 6 Research Institute of Agriculture and Life Sciences, Seoul National University, Seoul, 08826 Republic of Korea; 7 Nbiocell Inc, Siheung SNU Start-up Campus, Gyeonggi-do, 15011 Republic of Korea

**Keywords:** Nanoparticles, Membrane structure and assembly

## Abstract

Controlling the sizes of liposomes is critical in drug delivery systems because it directly influences their cellular uptake, transportation, and accumulation behavior. Although hydrodynamic focusing has frequently been employed when synthesizing nano-sized liposomes, little is known regarding how flow characteristics determine liposome formation. Here, various sizes of homogeneous liposomes (50–400 nm) were prepared according to flow rate ratios in two solvents, ethanol, and isopropyl alcohol (IPA). Relatively small liposomes formed in ethanol due to its low viscosity and high diffusivity, whereas larger, more poly-dispersed liposomes formed when using IPA as a solvent. This difference was investigated via numerical simulations using the characteristic time factor to predict the liposome size; this approach was also used to examine the flow characteristics inside the microfluidic channel. In case of the liposomes, the membrane rigidity also has a critical role in determining their size. The increased viscosity and packing density of the membrane by addition of cholesterol confirmed by fluorescence anisotropy and polarity lead to increase in liposome size (40–530 nm). However, the interposition of short-chain lipids de-aligned the bilayer membrane, leading to its degradation; this decreased the liposome size. Adding short-chain lipids linearly decreased the liposome size (130–230 nm), but at a shallower gradient than that of cholesterol. This analytical study expands the understanding of microfluidic environment in the liposome synthesis by offering design parameters and their relation to the size of liposomes.

## Introduction

Phospholipids play a critical role in nature as components of cell membranes^1,2^. In aqueous solutions, phospholipids form a bilayer structure due to their hydrophobic effects^3^; one such bilayer structure, called a liposome, is incredibly useful as it can encapsulate aqueous solutions, and therefore can be used to contain hydrophilic drugs^4,5^. Liposomes have thus been extensively investigated as nanocarriers for delivering exogenous molecules. Moreover, their application potential has been explored across a range of various fields, such as cosmetics^6,7^, agriculture^8,9^, medicine^10,11^, and food^12,13^ products. However, to strengthen their feasibility as a delivery system, it is important to be able to control the size distribution of liposomes^14^.

Liposomes are typically prepared via sonication, mechanical stirring, or extrusion methods^15–17^, none of which ensure the synthesis of monodispersed liposomes, nor do they offer size control. Moreover, biomolecules such as peptides or membrane proteins can be denatured by the external energy exerted during these manufacturing processes^18^. Therefore, there has recently been a shift away from these bulk methods for preparing liposomes, with attention now being focused on microfluidic methods^19–21^; these methods enable the flow to be controlled precisely, such that monodispersed, nano-sized liposomes can be synthesized. These microfluidic methods for preparing liposomes are classified by the type of solvent employed^22^. When using an immiscible solvent, water-in-oil-in-water (W/O/W) emulsion is used as a liposome template, with the oil phase being removed after emulsification using drag force and evaporation processes^23^. However, completely removing the oil phase is difficult, and this method has limitations regarding the reduction of liposome size.

In contrast, microfluidic methods that use miscible solvents induce the self-assembly of lipids by mixing the solvent and buffer and increasing the polarity^24^. Hydrodynamic focusing utilizes the diffusion characteristics of a solvent to develop uniform mixing through a microfluidic channel^25^. Moreover, the use of a thin solvent phase has been shown to enhance the mixing efficiency by focusing the inner stream. Therefore, hydrodynamic focusing has frequently been employed when synthesizing nano-sized liposomes. The dependency of liposome size on the total flow rate (TFR)^26^, flow rate ratio (FRR)^27^, temperature^24^, and lipid concentration^28^ have all been experimentally investigated. Moreover, techniques have been developed to enhance the mixing uniformity, such as three-dimensional (3D) hydrodynamic focusing^29^ or the thin jet co-flowing method^30^. Although hydrodynamic focusing has been extensively studied, to the best of our knowledge, there has been no quantitative investigation of how flow characteristics determine liposome formation, nor have there been any attempts to rationalize the observed gaps in results between experiments.

In the hydrodynamic focusing method, the width of inner stream determines the mixing characteristics in the microfluidic channel, which can be adjusted by TFR and FRR^28^. Especially, FRR significantly affects the inner stream width^26,28^ so that the correlation between FRR and liposome size has been investigated in the previous studies^27,31^. However, FRR is coupled to the total lipid concentration, which also affects the liposome size and thereby it is not clear that downsized liposome comes from the different mixing characteristics or decreased lipid concentration. In the present study, the effect of solvent, which is concentration-invariant, was investigated numerically and experimentally. The diffusional motion and stream width are dependent on the diffusivity and viscosity of the solvent being used so that the nucleation and growth of nanoaggregates are determined by the solvent properties as well as FRR^27,32^.

Meanwhile, the liposome nanoformulation requires the vesiculation process, implying that the membrane stiffness has an influence on the size of liposome in addition to the nucleation aspect, which was examined through the addition of two additives: cholesterol and short-chain lipids. Cholesterol is widely used to enhance membrane rigidity, while short-chain lipids may potentially degrade membranes by de-aligning their lipids^33–35^. Here, liposomes containing cholesterol and short-chain lipids were synthesized hydrodynamically, and their membrane properties were measured via spectroscopic methods and then analyzed in relation to liposome size.

## Results and discussions

**Figure 1:**
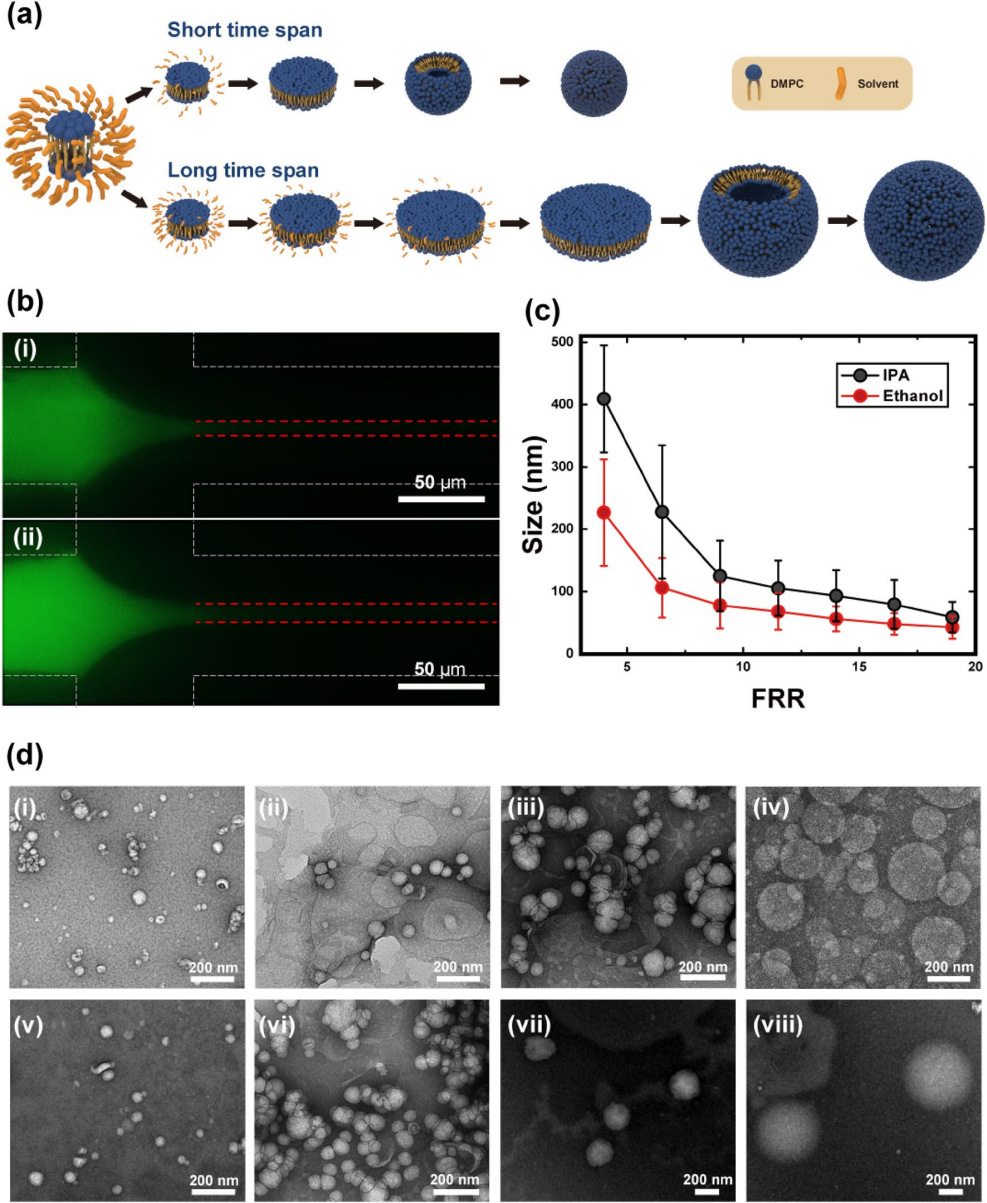
(**a**) Vesiculation of the bilayer domain according to the time span for self-assembly. Upper side represents a short time span case (corresponding to fast depletion of the solvent); lower side shows slow vesiculation over a longer time span. (**b**) Fluorescence image of a focused stream according to the solvent (total flow rate = 2000, flow rate ratio (FRR) = 9, (i) Ethanol, (ii) isopropyl alcohol (IPA), Scale bar = 50 µm). (**c**) Size of the liposome depending on the FRR for two different solvents. Red and black circles represent ethanol and IPA, respectively. (**d**) TEM images of the liposomes prepared using ethanol and IPA at (i, v) FRR = 19, (ii, vi) FRR = 11.5, (iii, vii) FRR = 6.5, and (iv, viii) FRR = 4 (Scale bar = 200 nm).

As shown in Fig. 1, liposome size decreased with increasing FRR, in agreement with previously reported results^28,36^. The morphologies of prepared liposomes were verified via transmission electron microscopy as described in Fig. 1d, which were coincident to DLS measurement data (58.92 ± 24.55 nm, 105.4 ± 44.28 nm, 227.8 ± 106.9, and 409.2 ± 85.91 nm at FRR = 19, 11.5, 6.5, and 4, respectively). Vesicles formed during the solvent-anti-solvent method via the following method. As the solvent content decreased via mutual diffusion with the buffer, monomeric lipids started to nucleate and form a bilayer disk due to the elevation of polarity. Vesiculation of this intermediate disk then occurred, provided that the disk’s length was large enough to allow it to bend over and close into a sphere, compared to its line tension. This balance between line tension and the rigidity of the intermediate disk is described as a vesiculation index, as shown in Eq. (1). This indicates that vesiculation depends on the length and bending modulus of the disk, and on the coverage of stabilization molecules^37^.

1 $${V}_{f}=\frac{r{\Lambda }_{0}}{4\kappa }\left[1+\frac{kT}{{\alpha }_{b}}ln\left(1-{\phi }_{r}\right)\right]$$

where ${V}_{f}$ is the vesiculation index, $r$ is the disk size, ${\Lambda }_{0}$ is the line tension, $\kappa$ is the bending modulus, ${\alpha }_{b}$ is the energy gain upon binding of one stabilization molecule, and ${\phi }_{r}$ is the fraction of the rim area covered by stabilization molecules.

Here, the stabilization molecule can be considered a solvent and, thus, the rapid depletion of the solvent would bring the disk into an unstable state, thereby accelerating vesiculation^24^. Furthermore, rapid, and uniform nucleation has been observed during a fast solvent depletion case, which downsized the synthesized assemblies^38,39^.

Consequently, the observed decrease in liposome size with increasing FRR resulted from the rapid depletion of the solvent owing to the shortened diffusion length^40,41^. This relationship can also explain the dependence of the size difference on the solvent type. The width of the solvent phase increases with increasing viscosity (of the solvent), as shown in Eq. (2). Thus, ethanol is more thinly focused than IPA (Fig. 1b)^41^.

2 $$\frac{\delta }{W}=\frac{1}{1+0.67{\mathrm{\varnothing }}^{-2/3}{\chi }^{-1/2}}$$

where $\delta$ is the stream width, $W$ is the channel width, $\varnothing$ is the FRR (${Q}_{outer}/{Q}_{inner}$), and $\chi$ is the viscosity ratio of the solvent and buffer (${\mu }_{solvent}/{\mu }_{buffer})$.

Furthermore, the diffusivity of ethanol is larger than that of IPA, indicating that ethanol diffuses out more rapidly. Moreover, smaller liposomes were synthesized by employing ethanol as the solvent, as shown in Fig. 1c. Consequently, the solvent depletion rate was positively correlated with the rate and uniformity of nucleation, and with the increasing instability of the intermediate disk. Thus, scenarios with a high FRR, or where ethanol was used as a solvent, exhibited a short-time span step, as shown in Fig. 1a; the other case displayed a long time-span step.

The kinetic model and nucleation theory offer a conceptual clue as to how the rate of solvent depletion could affect liposome size. However, in the hydrodynamic focusing method, the solvent content varies dynamically, and solvent molecules also participate in the nucleation and growth of the bilayer disk. The coalescence of the lipid bilayer is accelerated under low solvent concentrations^37^; therefore, the relationship between liposome size and solvent content must be treated as a coupled problem.

**Figure 2:**
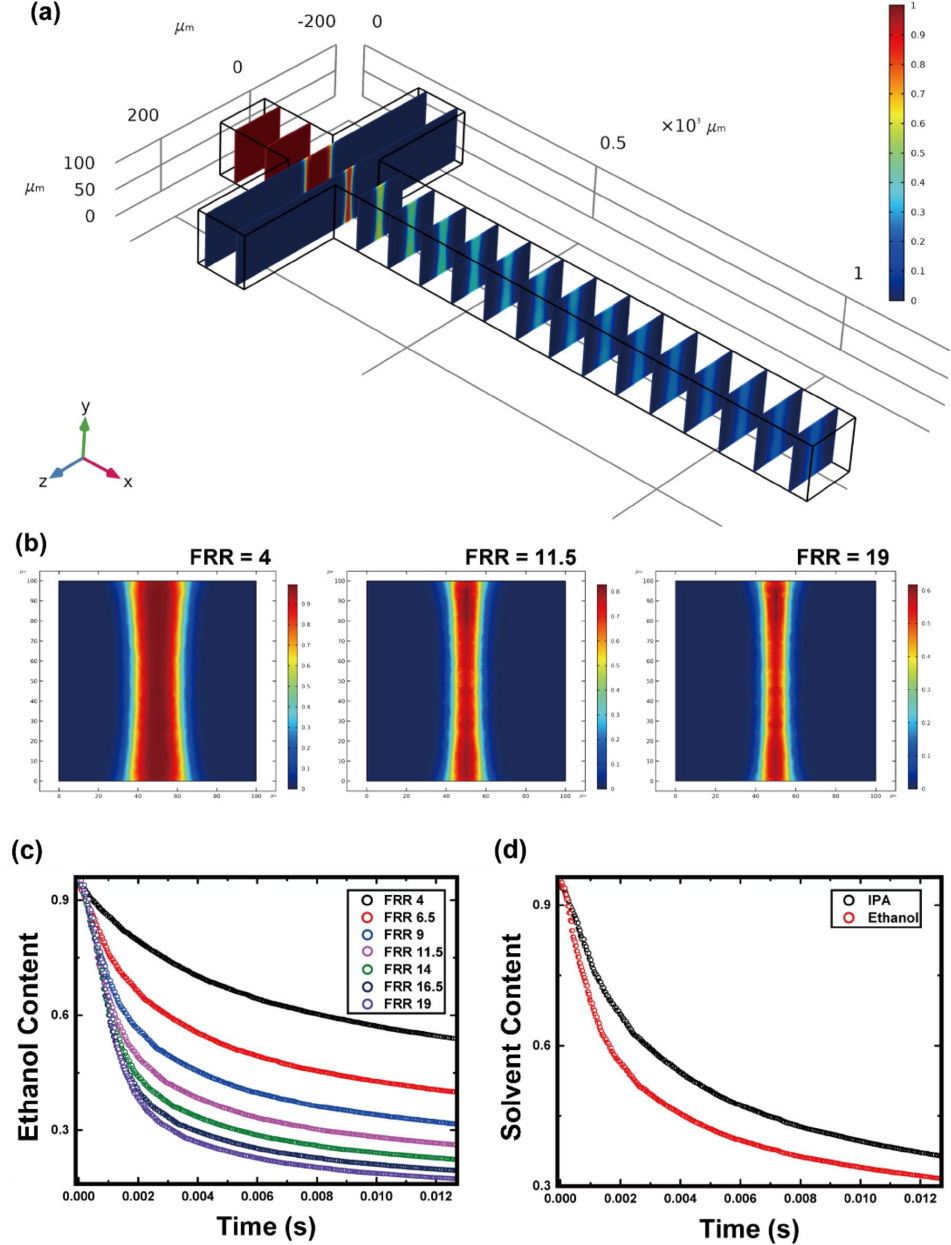
(**a**) Three-dimensional numerical simulation result for ethanol concentration at steady state. (**b**) Ethanol concentration at the yz-plane. Cut plane is placed at x = 250 µm, where the cross-junction end and long diffusive mixing section starts. Lagrangian content of the solvent over time is shown plotted in relation to the (**c**) flow rate ratio (FRR) and (**d**) solvent type. Whole data were processed by MATLAB R2019B (MathWorks, Inc., Natick, MA, USA) using Eqs. (3) and (4).

The diffusivity of lipids can be neglected because compared to solvent or buffer molecules, lipids do not significantly deviate the streamline of flow. Thus, the lipid molecules in this study were confined within the focused-solvent phase and the nucleation process commenced in the interfacial area where the mutual diffusion of solvent and buffer molecules occurred. As mentioned above, the simultaneity and uniformity of nucleation together largely influenced the assembly size by affecting the growth of the intermediate disk. This effect was negatively correlated with the volume of the solvent-residing phase. The amount of time taken for the solvent and buffer molecules to be fully exchanged was also found to be critical, suggesting that the solvent depletion profile at the centerline warranted further investigation. Therefore, numerical simulations were performed for 3D microchannels with cross junctions, as shown in Fig. 2a, from a Lagrangian perspective.

At the intersection plane where the diffusion-dominant mixing of solvent and buffer began, the solvent became more focused as the FRR increased (Fig. 2b). To verify the effect of the solvent content on lipid assembly, it was necessary to examine the solvent content by tracking the flow of the lipids along the channel. Thus, the solvent content was reorganized into the Lagrangian coordinate system by integrating the steady-state velocity field, as described in Eqs. (3) and (4):

3 $${C}^{*}=C(x={x}_{l}\left(t\right), y={y}_{l}\left(t\right), z={z}_{l}\left(t\right))$$

4 $${x}_{l}\left(t\right)={x}_{l,0}+\int u\left({x}_{l},{y}_{l},{z}_{l}\right)dt, {y}_{l}\left(t\right)={y}_{l,0}+\int v\left({x}_{l},{y}_{l},{z}_{l}\right)dt, {z}_{l}\left(t\right)={z}_{l,0}+\int w\left({x}_{l},{y}_{l},{z}_{l}\right)dt$$

where $u\left({x}_{l},{y}_{l},{z}_{l}\right)$ is the x-directional velocity at $\left({x}_{l},{y}_{l},{z}_{l}\right)$, $v\left({x}_{l},{y}_{l},{z}_{l}\right)$ is the y-directional velocity at $\left({x}_{l},{y}_{l},{z}_{l}\right)$, and w $\left({x}_{l},{y}_{l},{z}_{l}\right)$ is the z-directional velocity at $\left({x}_{l},{y}_{l},{z}_{l}\right)$.

Consequently, the solvent content according to the control volume flowing along the channel centerline is depicted in Fig. 2c,d and denominated as the Lagrangian content of the solvent. As shown in Fig. 2c, the solvent content decreased more rapidly as FRR increased, in line with the above hypothesis. Furthermore, ethanol was shown to diffuse more rapidly than IPA (Fig. 2d).

**Figure 3:**
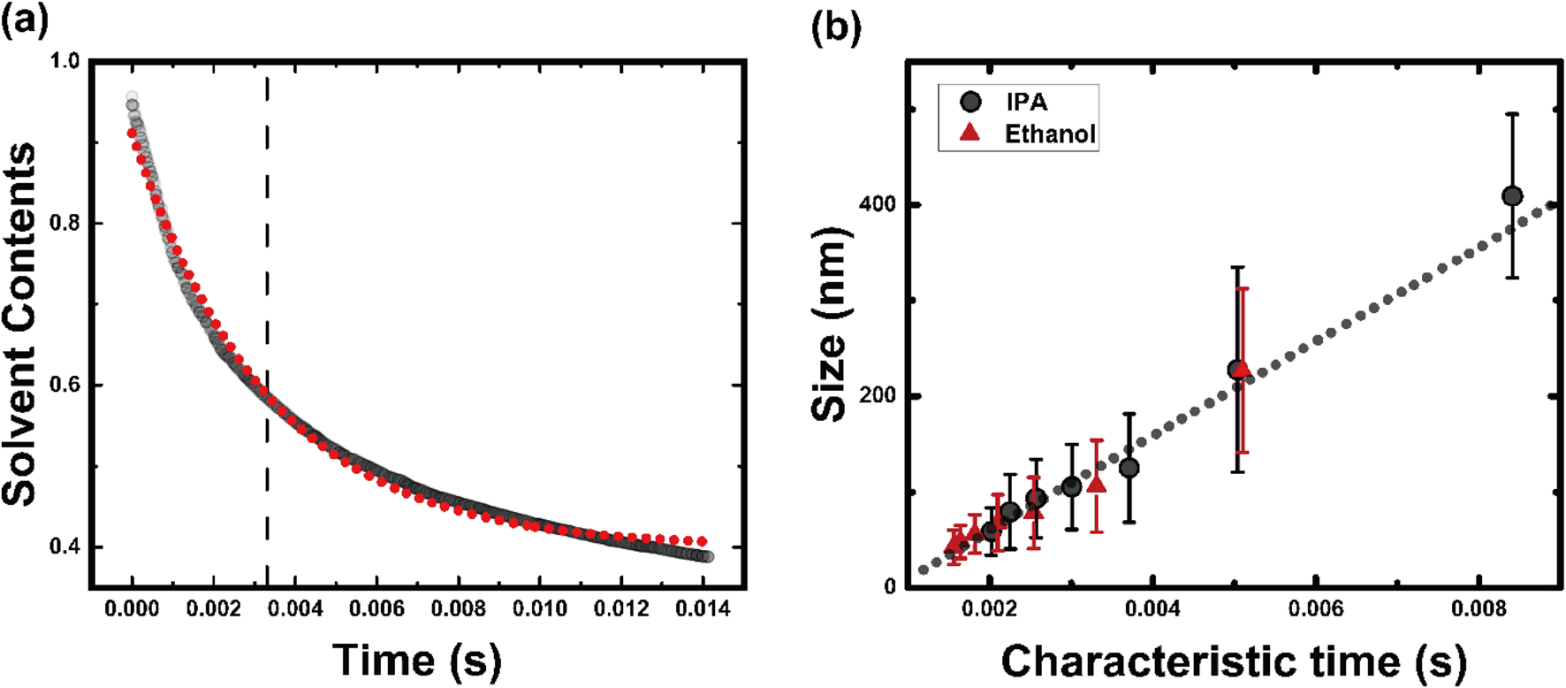
(**a**) Exponential fitting of the ethanol content. Red dots represent an exponentially fitted curve and dashed line indicates the decay constant. (**b**) Relationship between liposome size and characteristic time of self-assembly. Black dotted line represents a linear curve fitted using the least-squares method (${R}^{2}=0.98$) and red and black circles are experimental results for ethanol and IPA, respectively. Characteristic time was obtained from the decay constant of the exponentially fitted curve for the Lagrangian content of the solvent.

The depletion of the solvent at the centerline of the inner stream was approximated to follow an exponential decay function, in which a decay constant, λ, represented the time span between the start and end points of the domain growth; this is termed the characteristic time of self-assembly (Fig. 3a).

All FRR and solvent data were plotted in terms of the characteristic time of self-assembly, which was obtained numerically (Fig. 3b). The liposome size increased with increasing characteristic time. Moreover, this linear relationship held true even for different solvents (ethanol vs. IPA). Thus, the size of the intermediate domain, which determines the size of the liposome, could be properly described by the characteristic time, regardless of the solvent being used in the hydrodynamic focusing system.

**Figure 4:**
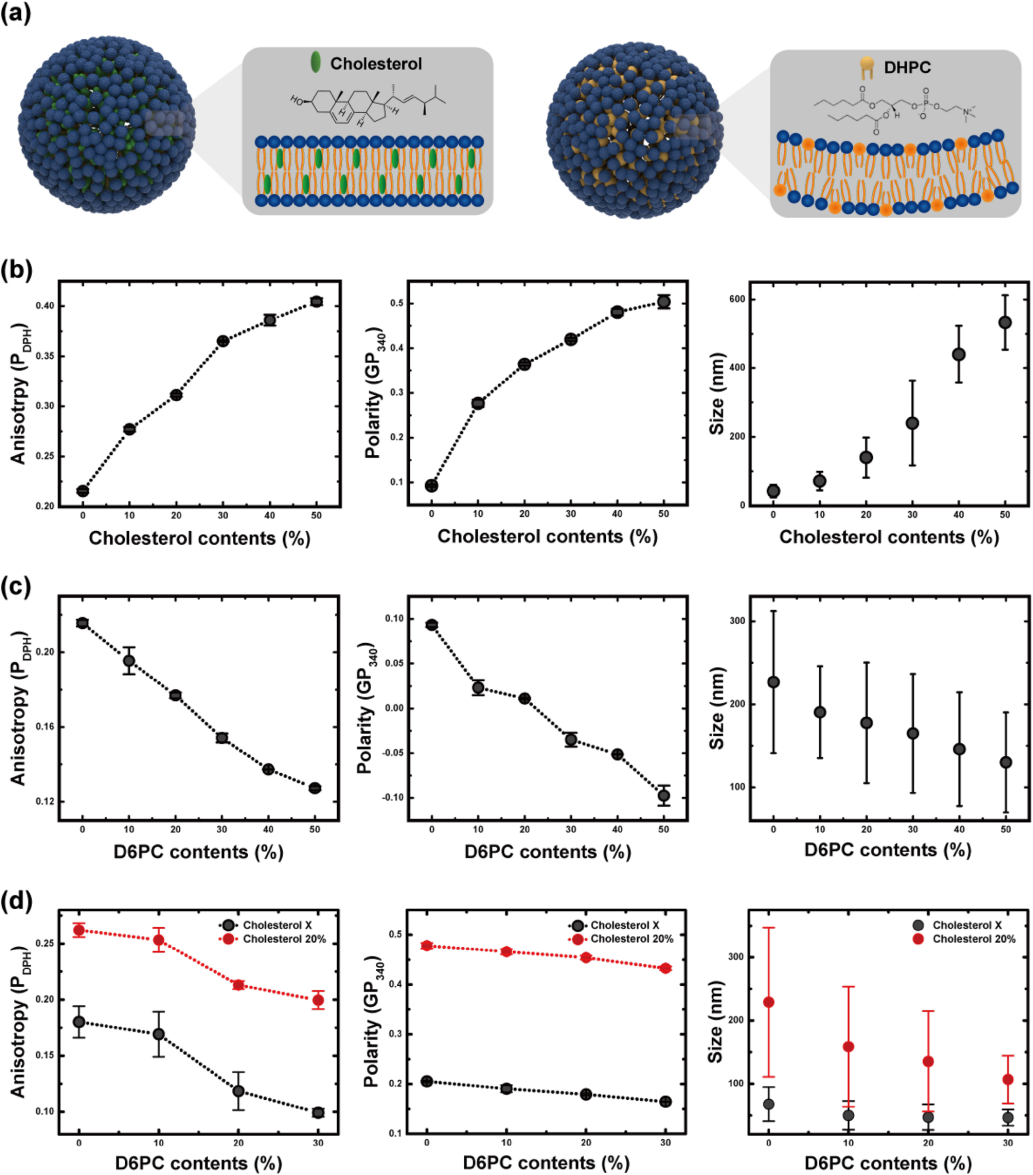
(**a**) Vesicle containing cholesterol and a heterogeneous vesicle containing short-chain lipids. Relationships between membrane properties (fluorescence anisotropy and polarity) and liposome size depending on (**b**) cholesterol content (flow rates of the ethanol and buffer phases fixed at 100 and 1900 µL h^−1^, respectively) and (**c**) 1,2-dihexanoyl-sn-glycero-3-phosphocholine (DHPC) content (flow rates of the ethanol and buffer phases fixed at 400 and 1600 µL h^−1^, respectively). (**d**) The membrane properties and size depending on DHPC contents when the ethanol content was reduced (flow rates of the ethanol and buffer phases fixed at 100 and 1900 µL h^−1^, respectively).

The liposome size is also dependent on the bending modulus, according to Eq. (1). A membrane with a high bending modulus (i.e., a rigid membrane) will have a high resistance to bending, so the growth of the bilayer domain will be maintained and vesiculation will be postponed. Thus, the liposome size was predicted here to be positively correlated to the membrane rigidity. To verify this relationship, cholesterol was added into the solvent phase, which is known to indurate the bilayer membrane (Fig. 4a). Furthermore, the effect of membrane orderliness (mediated by its heterogeneity) was also experimentally investigated by adding short-chain lipids.

Membrane rigidity was evaluated using two indicators: fluorescence anisotropy and polarization. The fluorescence anisotropy was measured using DPH as a fluorescence probe. The hydrophobicity and rod-like shape of DPH allow it to become oriented along lipids while being intromitted between hydrophobic chains. Thus, its motion mimics the rotational diffusion of lipid chains^42^. Therefore, high anisotropy value indicates that the rotational diffusion is hindered, which can be interpreted that the viscosity of the local environment of the membrane is elevated. As shown in Fig. 4b, the anisotropy increased as more cholesterol was added, indicating that cholesterol acted as a thickener for the membrane.

The membrane polarity was analyzed using the fluorescent dye, Laurdan, which can inform the degree of lipid packing. Laurdan is highly sensitive to the presence and mobility of a dipole. Therefore, it can reflect the extent of water molecules in its vicinity^43,44^. For an ordered-gel phase membrane, it is difficult for water molecules to penetrate the head groups of lipids, resulting in an increase of membrane polarity. Figure 4b shows that the membrane polarity increased following the addition of cholesterol, implying that the presence of cholesterol induced a dense packing and dehydration of the membrane.

Overall, the membrane properties were enhanced by adding cholesterol. A rigid membrane will preferentially exist in a planar state, rather than as a vesicle; this can delay vesiculation and enlarge the resulting liposome. As shown in Fig. 4b, the liposome size increased following the addition of cholesterol, which reflects the relationship between membrane rigidity and liposome size.

DHPC, which is also a phospholipid but has a short acyl chain (C6), unlike DMPC (C14), was added to de-align the bilayer membrane. A membrane containing DHPC molecules was predicted to be disordered due to its chain length difference, with such a degraded membrane causing early vesiculation and forming smaller liposomes. As shown in Fig. 4c, the addition of DHPC decreased fluorescence anisotropy and polarization, indicating that the heterogeneous membranes had degraded properties.

Likewise, the effects of cholesterol and DHPC were inversely related in this aspect. For a molar ratio of cholesterol to DMPC of 0.5, the increment of membrane polarity was 0.4; the decrement was less than 0.2 for the same molar ratio of DHPC. Fluorescence anisotropy showed similar trends, but the difference between the two additives was slightly lower, implying that the chain length difference presumably preserved more space for DPH to undergo diffusional motion, resulting in the observed decrease in anisotropy, but the packing of the bilayer was essentially maintained by the presence of DHPC molecules. Finally, as confirmed in Fig. 4c, the liposome size decreased linearly following the addition of DHPC, but the gradient was reduced compared to that for cholesterol. The structural stability of heterogeneous vesicles composed of DMPC and DHPC was relatively low, so the lubricating effect of DHPC was also studied when the membrane becomes rigid. Reducing the ethanol content and adding cholesterol improved the membrane properties, as expected, and the effect of DHPC was confirmed to be maintained, as shown in Fig. 4d.

**Figure 5:**
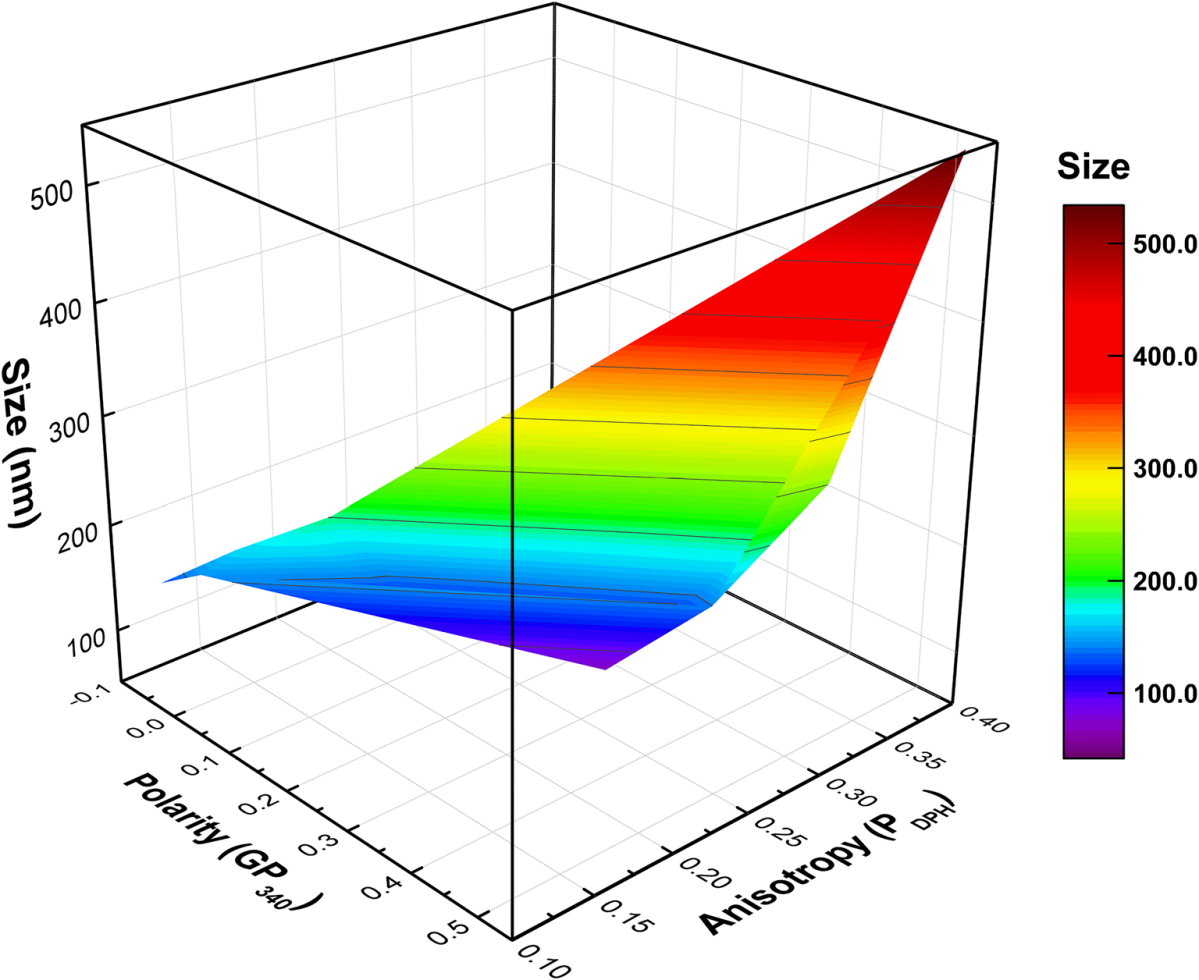
Response surfaces of liposome size as a function of membrane properties depending on the contents of the cholesterol and DHPC. The fluorescence anisotropy and polarity represent a local viscosity and packing density of the membrane, respectively. Liposome size increased as the anisotropy or polarity value increased.

Herein, a wide range of liposomes (42–534 nm) were synthesized in a hydrodynamic focusing system, according to the characteristic time and membrane properties. For pure vesicles, the liposome size was determined by the characteristic time. However, in scenarios where the membrane composition was changed, the membrane properties also influenced liposome size. Finally, the liposome size could be estimated using two factors representing the membrane properties, as shown in Fig. 5. Overall, the size of liposomes increased with high membrane polarity and anisotropy originated from the addition of cholesterols, while DHPC induced heterogeneity led to the decrease in the liposome size.

## Conclusions

Monodispersed nano-sized liposomes were synthesized in a microfluidic chip using hydrodynamic focusing, owing to the fast and uniform mixing of the solvent and buffer. Liposome size was precisely controlled by changing the FRR, with this relationship behaving differently depending on the solvent used. Using ethanol, which is a low viscosity and fast diffusing solvent, resulted in smaller sized liposomes than those prepared using IPA. This size change is presumably attributable to the different solvent depletion rates. The polarity in the vicinity of lipids flowing along the channel was found to be dependent on the Lagrangian solvent content, such that alternating the rate of decrease of the solvent content allowed enough time for growth to be changed. The characteristic time span from a solvent-rich to a buffer-rich circumstance was defined herein using a decay constant of the solvent content, which was numerically obtained by tracking the solvent content along lipids flowing through the channel. Using this newly defined parameter, the effects of the FRR and solvent on the liposome size could be interpreted by using this linear relationship to predict liposome sizes under different conditions. Moreover, the effect of membrane rigidity was investigated through the addition of two additives, cholesterol and DHPC. Adding cholesterol increased the rigidity of the membrane, which delayed vesiculation and caused a relatively large liposome to be synthesized. In contrast, adding DHPC molecules led the membrane to enter a fluid phase by de-aligning its lipids. This degradation of the membrane meant that smaller liposomes were synthesized, even though the total lipid concentration increased.

As such, the characteristic time of self-assembly and membrane rigidity were identified here as the main parameters affecting liposome size. For future liposome synthesis application, selecting solvents and lipid types or designing the microfluidic chip should be guided with respect to the results of this study.

Additional experiments using another solvent or other types of lipids might give further understanding of microfluidic production for liposome. The liposomes prepared using methanol is expected to be smaller considering that the viscosity of it is lower than ethanol or IPA, while the liposome prepared with long-chain lipids such as 1,2-dihexadecanoyl-sn-glycero-3-phosphocholine (DPPC) or 1,2-distearoyl-sn-glycero-3-phosphocholine (DSPC) might be larger than DMPC liposome because the phase transition temperature is high. Furthermore, it is noteworthy that the liposome size is also dependent to the TFR, which changes mixing characteristics of the microfluidic channel. However, a discussion of TFR effect was beyond the scope of this study, because increase of TFR leads to the elevation of Reynold’s number and the pressure toward channel centerline, which can originate unstable flow stream. We hope that further studies addressing whole parameters could be conducted to fully enable the precise prediction of liposome size in microfluidic systems; such a development could significantly broaden the feasibility of using liposomes in applied roles.

## Methods

### Materials and reagents

SU-8 2100 negative photoresist (PR) was purchased from MicroChemicals GmbH (Ulm, Germany), and an Si wafer (525-μm thick) was used to fabricate a silicon master mold. Polydimethylsiloxane (PDMS, Sylgard184, mixed with a 10:1 ratio of curing agent) for soft molding was purchased from Dow Corning (Midland, MI, USA); 1,2-dimyristoyl-sn-glycero-3-phosphocholine (DMPC, C 14:0, ${\mathrm{T}}_{\mathrm{m}}$ = 23 °C) and 1,2-dihexanoyl-sn-glycero-3-phosphocholine (DHPC, C 6:0) were purchased from Avanti Polar Lipids, Inc. (Alabaster, AL, USA). Phosphate-buffered saline (PBS), cholesterol, 1,6-diphenyl-1,3,5-hexatriene (DPH), and 6-dodecanoyl-N,N-dimethyl-2-naphthylamine (Laurdan) were obtained from Sigma-Aldrich (St. Louis, MO, USA) and used to analyze membrane properties (fluorescence anisotropy and polarity). Calcein was purchased from Tokyo Chemical Industry co., LTD (Tokyo, Japan).

### Fabrication of a microfluidic chip

A silicon master mold was fabricated according to the general procedures of photolithography for negative PR (SU-8 2075)^45^. The PR was uniformly spin-coated to a thickness of 80 μm and then baked at 60 °C for 3 min and 95 °C for 9 min. The hardened PR was then exposed to ultraviolet light at an intensity of 10 mJ s^−1^ using a mask aligner. Post-exposure baking was completed at 60 °C for 2 min, followed by 95 °C for 9 min. To remove the residual PR, an Si wafer was developed for 5 min.

To fabricate each microfluid chip, a base polymer and a curing reagent were mixed at a ratio of 10:1, cast onto the Si master mold, and then degassed in a vacuum chamber. The degassed PDMS was baked at 70 °C in a curing oven for 2 h and then carefully detached from the Si master mold. The PDMS microfluidic channel was sealed by binding the microfluidic patterned plate to the flat PDMS plate after plasma treatment. The bonded chip was then cured at 80 °C in a convection oven for 8 h^46^.

**Figure 6:**
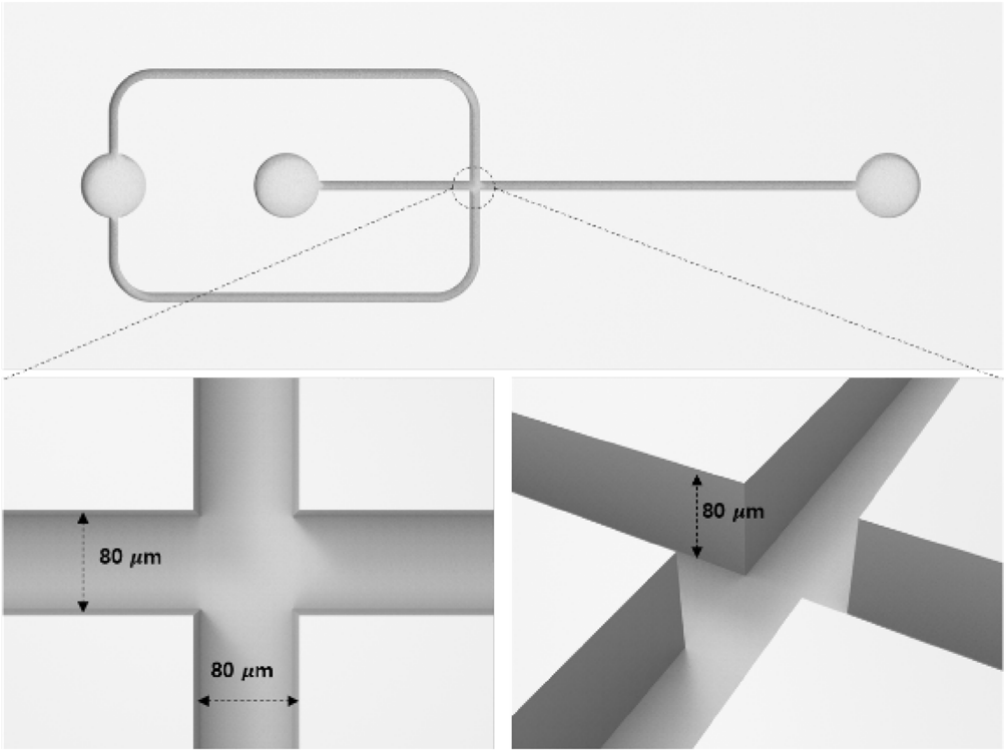
Illustration of the microfluidic chip employed to prepare the liposomes.

The dimensions of the PDMS microfluidic chip were 11 mm × 35 mm × 8 mm. The channel was composed of a cross-junction with two inlets and an outlet, and the width and height of the channel were both 80 μm (aspect ratio = 1). The microchannel was plane type without any microstructures, such as herringbone structures, as shown in Fig. 6.

### Liposome preparation

Liposomes were synthesized using a cross-junction microfluidic chip, where solvents containing DMPC (20 mM) and PBS (10 mM) were employed as the inner and outer phases, respectively. At the cross-junction, the inner and outer phases met and mixed through mutual diffusion. The flow rate was adjusted using two syringe pumps with a fixed total flow rate of 2000 µL h^−1^ for the consistent and sufficiently low Reynold’s number condition; the buffer-to-solvent FRRs were also varied. After the focused stream was stabilized, 10 mL of each sample was collected into glass vials and kept in the refrigerator for further analysis. All samples were prepared at 25 °C which is above the phase transition temperature of DMPC.

### Size characterization by dynamic light scattering

The sizes of vesicles were determined by the distribution analysis using a non-negatively least squares (NNLS) fitting algorithm of dynamic light scattering measurements (Zetasizer Nano ZS90; Malvern Panalytical Ltd, Malvern, UK)^47^. The cumulant size data are also shown in the supporting information (see Table S1). In detail, 1 mL of sample solution was loaded into a quartz cell to prevent aggregation, and scattered light intensity was measured at 20 °C without any sample dilution. The hydrodynamic radius was determined by the Stokes–Einstein equation (Eq. 5), as follows:

5 $${r}_{h}= \frac{kT}{6\pi \eta D}$$

where *k* is the Boltzmann constant, *T* is the absolute temperature, *η* is the viscosity of the sample, and *D* is the diffusion coefficient.

### Characterization of membrane properties

The fluidity of a lipid membrane was evaluated with a fluorescent probe (i.e., DPH)^48^. DPH was dissolved in a lipid solution at a molar ratio of 1:250. DPH molecules were inserted into the lipid membrane and accumulated at the middle of the bilayer. The fluorescence anisotropy of DPH was measured using a fluorescence spectrophotometer (SCINCO, FS-2). The DPH-loaded lipid solution was excited at 360 nm, and emission intensities were measured at 430 nm. The membrane fluidity (${1/\mathrm{P}}_{\mathrm{DPH}}$) equation is as follows:

6 $$\frac{1}{{P}_{DPH}}= \frac{{I}_{\parallel } + G{I}_{\perp }}{{I}_{\parallel } - G{I}_{\perp }} (G= {i}_{\perp } / {i}_{\parallel })$$

where ${\mathrm{I}}_{\parallel }$ and ${I}_{\perp }$ represent emission intensities parallel and perpendicular to horizontally polarized light, respectively; and ${\mathrm{i}}_{\parallel }$ and ${\mathrm{i}}_{\perp }$ represent emission intensities parallel and perpendicular to vertically polarized light, respectively. Given that the freely diffused DPH molecules can be rotated between lipid bilayers, membrane fluidity can be evaluated in combination with polarized intensities at multiple angles^49^.

The polarity of a lipid membrane was evaluated with a fluorescent probe (i.e., Laurdan)^50^. Laurdan was dissolved in a lipid solution at a molar ratio of 1:200. Laurdan molecules were inserted into the edge of the lipid membrane surface and accumulated on the membrane surface. The general polarization of Laurdan was measured using a fluorescence spectrophotometer (SCINCO, FS-2). Laurdan-loaded lipid solution was excited at 340 nm, and emission intensities were measured at 440 and 490 nm. The membrane polarity (${\mathrm{GP}}_{340}$) equation is as follows:

7 $${GP}_{340}= \frac{{I}_{440}- {I}_{490}}{{I}_{440}+ {I}_{490}}$$

where ${\mathrm{I}}_{440}$ and ${\mathrm{I}}_{490}$ represent emission intensities of 440 and 490 nm excited with 340 nm light, respectively. According to lipid membrane density, the emission spectrum of Laurdan can be shifted, and thus the $\mathrm{GP}$ value can be changed according to lipid membrane phase^44^.

### Numerical simulation method

A 3D model was created using COMSOL Multiphysics 5.5 (COMSOL, Inc., Burlington, MA, USA) to obtain concentration and velocity profiles at a steady state. Three-dimensional Navier–Stokes equations for incompressible fluid were used along with the continuity equation throughout the simulation domain; whole boundaries were used, at which a no-slip boundary condition was applied. Constant velocity values correspondent to the experimental conditions and atmospheric pressure were specified at the inlet and outlet, respectively. For stationary conditions, the Navier–Stokes and continuity equations were employed as shown in Eqs. (8) and (9), respectively:

8 $$\nabla \cdot \mathbf{u}=0$$

9 $$\uprho \mathbf{u}\cdot \nabla \mathbf{u}=-\nabla \mathrm{p}+\upmu {\nabla }^{2}{\varvec{u}}$$

where **u** is the velocity vector, p is the pressure, $\uprho$ is the density, and $\upmu$ is the dynamic viscosity.

Here, the density and viscosity are dependent on the concentration of solvent; therefore, they could be deduced using the rule of mixture^51^. The density and viscosity of solvents employed for the numerical simulation were described Table 1. The steady-state behavior of the solvent concentration was described by the stationary convection–diffusion equation (Eq. 10), as follows:

**Table 1:** Viscosity and density at 25 °C of each solvent.

Solvent type	Viscosity (mPa s)	Density (kg/m^3^)
Ethanol	1.040	786
IPA	1.960	789

Each property was obtained by built-in database in COMSOL Multiphysics 5.5.

10 $$\mathbf{u}\cdot \nabla \mathrm{C}=\mathrm{D}{\nabla }^{2}C$$

After the coupled system of the momentum transfer and mass transfer were iteratively solved inside the domain, the concentration and velocity profile could finally be obtained.

### Transmission electron microscopy

To verify the assembled shape of synthesized liposomes and validate DLS measurement results, the morphology of samples was investigated via transmission electron microscopy. 10 μL of each sample was transferred to a formvar/carbon 200 mesh copper grid (Electron Microscopy Sciences, Hatfield, PA, USA) and left for 5 min. The copper grid was then placed on 1% aqueous uranyl acetate drop for 1 min. After the staining process, the excess solution and stain were removed from the grid using filter paper. TEM images were obtained with Energy-Filtering Transmission Electron Microscope at 120 kV (Talos L120C, FEI, Czech).

### Data analysis

All the experimental data were expressed as mean ± standard deviation. The numerically obtained results were reorganized according to Eqs. (3) and (4) using in-house functions via MATLAB R2019B (MathWorks, Inc., Natick, MA, USA). Curve-fitting and data visualization were processed using Origin Lab software (Origin Pro 8, OriginLab Corp., USA).

The size distribution data were processed using both the cumulant and NNLS method, utilizing the built-in procedure of the Malvern Zetasizer (Malvern Panalytical Ltd, Malvern, UK).

### Supplementary Information

Supplementary Information.

## Data Availability

The authors confirm that the data supporting the findings of this study are available within the article. Raw data are available from the corresponding authors, upon reasonable request.
