# Author Correction: Precise control of liposome size using characteristic time depends on solvent type and membrane properties

**DOI:** 10.1038/s41598-024-55878-w

**Published:** 2024-03-04

**Authors:** Sunghak Choi, Bongsu Kang, Eunhye Yang, Keesung Kim, Moon Kyu Kwak, Pahn-Shick Chang, Ho-Sup Jung

**Affiliations:** 1 Present Address: Center for Food and Bioconvergence, Department of Food Science and Biotechnology, Seoul National University, Seoul, 08826 South Korea; 2 School of Mechanical Engineering, Kyungpook National University, Daegu, 41566 South Korea; 3 Department of Agricultural Biotechnology, Seoul National University, Seoul, 08826 Republic of Korea; 4 Research Inst. of Advanced. Materials, Collage of Engineering, Seoul National University, Seoul, 08826 South Korea; 5 Center for Agricultural Microorganism and Enzyme, Seoul National University, Seoul, 08826 Republic of Korea; 6 Research Institute of Agriculture and Life Sciences, Seoul National University, Seoul, 08826 Republic of Korea; 7 Nbiocell Inc, Siheung SNU Start-up Campus, Gyeonggi-do, 15011 Republic of Korea

Correction to: *Scientific Reports* 10.1038/s41598-023-31895-z, published online 23 March 2023

The original version of this Article contained an error in the Acknowledgements section.

“This work was supported by Korea Institute of Planning and Evaluation for Technology in Food, Agriculture and Forestry(IPET) through Agro and Livestock Products Safety·Flow Management Technology Development Program and High Value-added Food Technology Development Program, funded by Ministry of Agriculture, Food and Rural Affairs(MAFRA)(118105-3, 122029-4) and this work was partially supported under the framework of international cooperation program managed by the National Research Foundation of Korea(NRF-2020R1I1A1A01075049, NRF-2021R1I1A1A01059669) and this work was partially supported by the Bridge program by the Korea Environmental Industry &Technology Institute (2021002800015), which was funded by the Korean government.”

now reads:

“This work was supported by Korea Institute of Planning and Evaluation for Technology in Food, Agriculture and Forestry(IPET) through Technology Commercialization Support Program, and High Value-added Food Technology Development Program, funded by Ministry of Agriculture, Food and Rural Affairs(MAFRA) (821033-3, 122029-4) and this work was partially supported under the framework of international cooperation program managed by the National Research Foundation of Korea(NRF-2020R1I1A1A01075049, NRF-2021R1I1A1A01059669) and this work was partially supported by the Bridge program by the Korea Environmental Industry &Technology Institute (2021002800015), which was funded by the Korean government."

The original Article has been corrected.

