# Supplementary material for: Precise control of liposome size using characteristic time depends on solvent type and membrane properties
Source: Sci Rep. 2023 Mar 23;13:4728. doi: 10.1038/s41598-023-31895-z (PMC10036480; doi:10.1038/s41598-023-31895-z)
Supplement: Supplementary file 1 — Supplementary Information. [file 41598_2023_31895_MOESM1_ESM.docx]

**Supplementary information**

**Precise control of liposome size using characteristic time depends on solvent type and membrane properties**

Sunghak Choi^1,⸸^, Bong Su Kang^2,⸸^ , Eunhye Yang^3^, Kee Sung Kim^4^, Moon Kyu Kwak^2,*^, Pahn-Shick Chang^1,3,5,6,*^, and Ho-Sup Jung^1,*^.

^1^Center for Food and Bioconvergence, Department of Food Science and Biotechnology, Seoul National University, Seoul 08826, South Korea

^2^School of Mechanical Engineering, Kyungpook National University, Daegu 41566, South Korea

^3^Department of Agricultural Biotechnology, Seoul National University, Seoul 08826, Republic of Korea

^4^Research Inst. of Advanced. Materials, Collage of Engineering, Seoul National University, Seoul 08826, South Korea

^5^Center for Agricultural Microorganism and Enzyme, Seoul National University, Seoul 08826, Republic of Korea

^6^Research Institute of Agriculture and Life Sciences, Seoul National University, Seoul 08826, Republic of Korea
[^*^corresponding.author@email.example](mailto:*corresponding.author@email.example)

E-mail: mkkwak@knu.ac.kr (M.K.K), pschang@snu.ac.kr (P.-S.C), jhs@snu.ac.kr (H.-S.J)

**Zeta potential**

The zeta potentials of prepared liposomes were determined in Zetasizer nano ZS 90 (Malvern Panalytical Ltd, Malvern, UK). In detail, 1 mL of liposome sample was loaded into a quartz cell to prevent aggregation, and the zeta potential was measured at 20 °C without any sample dilution.

Phospholipid including DMPC has two charged group: phosphate group (anionic) and ammonium group (cationic) so that it is known to be zwitterionic. Thereby, as confirmed in Figure S1, the zeta potentials were shown to be around zero but slightly negative, overall. These slight negativities were observed in the previous study, which is attributed to the water molecules oriented and attached by hydrogen bonding to the lipids ^1^.





**Figure S1.** The zeta potential values of liposomes prepared using IPA or Ethanol. All experiments were performed in triplicate. All data were expressed as the mean value ± standard deviation.

**Cumulant size data of prepared liposomes**

Table S1. Detailed DLS data determined by cumulant analysis.

| **Solvents** | **Measurements** | **Flow Rate Ratio** | | | | | | |
| --- | --- | --- | --- | --- | --- | --- | --- | --- |
|  |  | **4** | **6.5** | **9** | **11.5** | **14** | **16.5** | **19** |
| **EtOH** | **Z-average (nm)** | 190.4 | 86.6 | 64.36 | 56.29 | 48.01 | 55.03 | 38.28 |
|  | **PDI** | 0.147 | 0.154 | 0.154 | 0.182 | 0.141 | 0.362 | 0.217 |
|  |  |  |  |  |  |  |  |  |
| **IPA** | **Z-average (nm)** | 382.3 | 188.9 | 101.5 | 85.36 | 75.09 | 63.56 | 52.67 |
|  | **PDI** | 0.433 | 0.239 | 0.182 | 0.184 | 0.178 | 0.238 | 0.201 |

References

1. Dreier, L. B. *et al.* Unraveling the Origin of the Apparent Charge of Zwitterionic Lipid Layers. *Journal of Physical Chemistry Letters* **10**, 6355–6359 (2019).
